# A tri-tuple coordinate system derived for fast and accurate analysis of the colored de Bruijn graph-based pangenomes

**DOI:** 10.1186/s12859-021-04149-w

**Published:** 2021-05-27

**Authors:** Jindan Guo, Erli Pang, Hongtao Song, Kui Lin

**Affiliations:** State Key Laboratory of Earth Surface Processes and Resource Ecology, Ministry of Education Key Laboratory for Biodiversity Science and Ecological Engineering, College of Life Sciences, Beijing Normal University, Beijing, China

**Keywords:** Genome graph, Coordinate system, Variant detection

## Abstract

**Background:**

With the rapid development of accurate sequencing and assembly technologies, an increasing number of high-quality chromosome-level and haplotype-resolved assemblies of genomic sequences have been derived, from which there will be great opportunities for computational pangenomics. Although genome graphs are among the most useful models for pangenome representation, their structural complexity makes it difficult to present genome information intuitively, such as the linear reference genome. Thus, efficiently and accurately analyzing the genome graph spatial structure and coordinating the information remains a substantial challenge.

**Results:**

We developed a new method, a colored superbubble (*cSupB*), that can overcome the complexity of graphs and organize a set of species- or population-specific haplotype sequences of interest. Based on this model, we propose a tri-tuple coordinate system that combines an offset value, topological structure and sample information. Additionally, *cSupB* provides a novel method that utilizes complete topological information and efficiently detects small indels (< 50 bp) for highly similar samples, which can be validated by simulated datasets. Moreover, we demonstrated that *cSupB* can adapt to the complex cycle structure.

**Conclusions:**

Although the solution is made suitable for increasingly complex genome graphs by relaxing the constraint, the directed acyclic graph, the motif cSupB and the *cSupB* method can be extended to any colored directed acyclic graph. We anticipate that our method will facilitate the analysis of individual haplotype variants and population genomic diversity. We have developed a C +  + program for implementing our method that is available at https://github.com/eggleader/cSupB.

**Supplementary information:**

The online version contains supplementary material available at 10.1186/s12859-021-04149-w.

## Introduction

### Background

Within a certain species, individual genomes vary in both the gene content and genomic portions of DNA sequences. How to accurately and comprehensively identify the genome-wide diversity of the species remains challenging. Recently, because of the rapid development of accurate long-read sequencing and assembly technologies [1, 2], for many species, abundant high-quality chromosome- and haplotype-resolved assemblies of species- or population-specific genomes have been derived, thereby accelerating the coming of the population genome era [3]. Together with various existing reference genomes, this type of genomic data has spawned a new research field called computational pangenomics [4]. The most accepted definition of pangenome is any collection of genomic sequences to be analyzed jointly or used as a reference [5]. A pangenome provides a complete picture of genomes and complex genomic variants within a species of interest and provides an opportunity for the development of efficient computational methods and various promising applications in medical biology [6], ecology [7], and evolutionary biology [8].

The graph-based model is among the most important representative models for the pangenome [5, 9]. Due to its good mathematical properties and theoretical foundation, graphs have achieved essential long-term status in biological sequence analysis [10–12]. The graph achieves compression and removal of redundancy and retains the continuity between sequences such that each sequence is encoded as a walk on the graph [13]. To better analyze the various visible or potential information provided by the graph, many basic and interesting topics, including graph selection, construction, storage, searching, mapping, and comparison, have been studied [14–18], but many challenges remain. One is to design appropriate coordinate systems for accurate graph spatial structure analysis. Defining a genome graph coordinate system that determines the correspondence between graph topology and sequence information is highly important, such as maintaining the translation between linear reference assemblies and genome graphs and improving annotations between existing assemblies and genome graphs [19].

The current coordinate systems can be roughly divided into two categories. The first type is one-tuple representation, which is based on the idea of ​​linearity. The basic research ideas include blocking, sorting, and constructing a graph according to genome alignments [20, 21]. However, this approach is an NP-hard problem and changes the topological structure of the graph while adding linearity, which inevitably leads to a loss of information. The second type is the multiple-tuple representation. For example, Rand et al. defined an offset-based coordinate system consisting of a region identifier and an offset [19]. One shortcoming of this approach is that it is suitable mainly for small sample sizes and simple variants. Most recently, Li H et al. designed a new system that combines a segment coordinate system with a binary group (segId; segOffset) on the sequence graph [22]; however, the topological information of the graph is not adequately described by these two methods.

### Our contribution

In this work, we designed a tri-tuple coordinate system for a class of colored de Bruijn graphs constructed from a set of genomes within a species by selecting an adequate k-mer length. We developed an efficient algorithm to identify each specific graph spatial structure, called a colored superbubble (cSupB), and organized these cSupBs into a tree that accurately reflects their inclusion relationships depicted in the colored de Bruijn graph we constructed. Compared to superbubbles [23] and ultrabubbles [24], each cSupB inherits similar advantages, such as globally organizing the sites without the existing reference genome. More importantly, the tri-tuple coordinate system is derived mainly from the inferred cSupB tree. To demonstrate the feasibility and efficiency of the approach, we elucidated the algorithm’s practical performance through a comprehensive set of experiments: (1) a real dataset of 12 human mitogenomes and (2) 310 simulated human mitogenome datasets. The results show that, on the one hand, the tri-tuple coordinate system can accommodate the existing linear reference coordinates and annotation data; on the other hand, the variants detected from the cSupBs are more comprehensive and diverse. Furthermore, we demonstrated that our analysis method is capable of managing tremendous amounts of genomes (5 k/20 k samples tested) with less memory and time consumption.

### Notation and related work

#### Representation

**Fig. 1:**
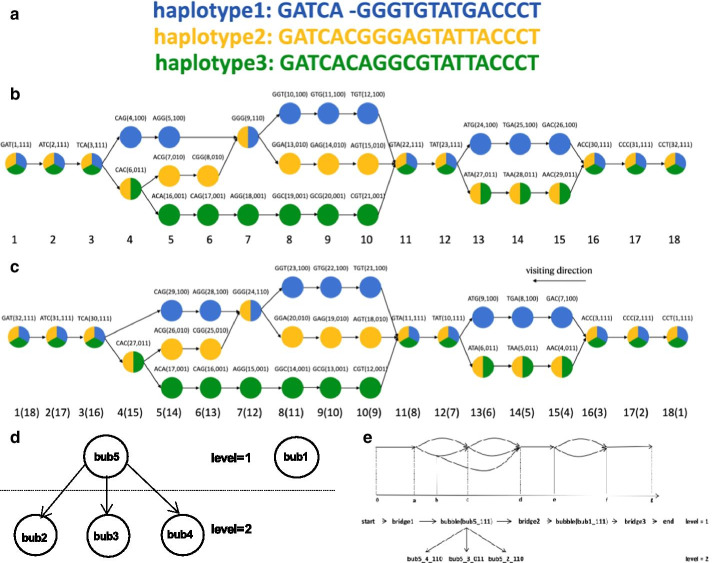
Schematic diagram for the construction, decomposition and reorganization of the colored de Bruijn graph. **a** The three original sequences. **b **The colored de Bruijn graph with k equal to 3. Each colored circle represents a node, and the black arrows represent edges. The characters above the node represent each node's label, visit order and color. The number below node represents the theoretical offset value of each node. $\mathbf{c}$ The result of visiting against the direction of the edge. The numbers below the node represent the final offset value and preoffset value. $\mathbf{d}$ The cSupB tree structure. Here, we find five cSupBs in the following order: bub1.$<\mathrm{TAT},\mathrm{ACC},111 >$; bub2.$<\mathrm{GGG},\mathrm{GTA},110>$; bub3.$<\mathrm{CAC},\mathrm{GTA},011>$; bub4.$<\mathrm{TCA},\mathrm{GGG},110>$; and bub5.$<\mathrm{TCA},\mathrm{GTA},111>$. $\mathbf{e}$ The final decomposition and representation of the colored de Bruijn graph

As discussed above, the colored de Bruijn graph, an extension of the classic de Bruijn graph, is essential for our algorithm. Given a set of sequences and a k value, we can transform the sequence into k-mers (unique strings of length k). Two different k-mers are adjacent if the k-1 base suffix of the “from” k-mer and the k-1 base prefix of the “to” k-mer are the same. If we regard each k-mer as a node and the adjacent relationship between k-mers as an edge, we can obtain a de Bruijn graph. By coloring the nodes and edges in the graph by the samples, we can extend classical de Bruijn graphs to the colored de Bruijn graph, as shown in Fig. 1. We provide the following definitions in the directed graph for the convenience of research.

- **Path**: A path from ${v}_{0}$ to ${v}_{k}$ refers to a sequence ${v}_{0},{e}_{1},{v}_{1},{e}_{2},\dots ,{e}_{k},{v}_{k}$. ${e}_{i}$ is the edge connecting ${v}_{i-1}$ with ${v}_{i}$, and the length of the path is k. If there is a path in the graph with the same start and end node, the path is "closed", indicating that the graph has a **cycle**.
- **Incoming node/edge**: For any two distinct nodes $u,v$, $u$ is called the **incoming node** of $v$ if there is a path from $u$ to $v$. The edges on this path are called the **incoming edges** of $v$, and the number of adjacent incoming edges is called the **indegree** of $u$.
- **Outgoing node/edge**: Based on the above definition, conversely, $v$ is called the **outgoing node,** and all the edges on the path are called the **outgoing edges** of $u$. Similarly, the number of adjacent outgoing edges is called the **outdegree** of $u$.
- **Degree**: The sum of the indegree and outdegree of a node is called the node’s **degree**.
- **Supernode**: If the outdegree or indegree of a node is greater than one, then the node is called a **supernode**.
- **Branch**: In this paper, a maximal path is called a branch if the outdegree and indegree of all nodes on the path are equal to 1.
- **Bridge**: *In this paper, a branch is called a bridge; if this branch is deleted, the number of connected components of the graph will increase.*
- **Bubble**: the structure formed by two paths redundant if they start and end at the same nodes is called a bubble [25]; for a general definition, see Definition 1 of superbubble in the cSupB subsection.

## Construction

Iqbal et al. first introduced the colored de Bruijn graph aimed at “detecting and genotyping simple and complex genetic variants in an individual or population” [26, 27]. As the space-efficient representations of de Bruijn graphs have also been heavily researched [28–31], many succinct data structures for the colored de Bruijn graph were developed and related software mainly includes the bloom filter trie (BFT) [32],VARI [33], rainbowfish [34], pufferfish [35], mantis [36], and bifrost [37]. In this paper, we use the VARI-merge [38], the new VARI version, to construct the colored de Bruijn graph. Given the large-scale samples and large size of the sequence data involved in whole genomes, VARI-merge can store and traverse genomes in a space- and time-efficient manner. In fact, the choice of the software is not particularly important because our study focuses on the analysis of graph structure instead of construction.

The colored de Bruijn graph construction includes four steps: k-mer extraction and counting, k-mer sorting, construction of the de Bruijn graph, and construction of the color matrix. The detailed installation process and usage method can be found at https://github.com/cosmo-team/cosmo/tree/VARI.

### Roadmap

In the next section, we describe the method from graph construction to decomposition and reorganization to applications including variant detection and coordinate system construction. Section 3 then elucidates the practical performance of the tri-tuple coordinate system and variant detection method. Section 4 offers some concluding remarks.

## Method

Our data structure for colored de Bruijn graphs is the most important research object in this paper, so we start by describing its construction. We then give a detailed explanation of how we traverse, decompose and reorganize the graph because graph spatial structure analysis is the basis of further research. Using analytical results, on the one hand, we propose a variant detection method for small indels; on the other hand, we design a tri-tuple coordinate system with details of our implementation.

### Graph construction

Let $\{{s}_{1},{s}_{2},\dots ,{s}_{n}\}$ be a set of haplotype genomic sequences. Because degeneracy is intractable for VARI, we replaced degenerate bases with the most frequent base of other sequences and recorded the modification. In addition, to ensure that each sequence does not have repeat k-mers, we find the longest repeat segment length ${k}_{i}$ of each sequence ${s}_{i}$ and take $K=max\{{k}_{1},{k}_{2},\dots ,{k}_{i}\}$. When the parameter k value is greater than $K$, the colored de Bruijn graph is almost guaranteed to be acyclic. Additionally, we randomly generate two segments consisting of ACGT with a length of k and add them to the front and end of each sequence to guarantee that the graph has only one start node and end node. The segment’s function is only to anchor the sequences, which does not affect the spatial framework, so it cannot appear in any original sequences. The segments are not fixed and change with the samples.

Finally, the obtained colored de Bruijn graph has the following characteristics: directed, acyclic, nondegeneracy, and unique start and end nodes. Moreover, each sequence corresponds to a unique path from the start node to the end node in the graph (Additional file 1: property 1; all the proofs of the properties, theorems, and corollaries can be found in Additional file 1).

### Decomposition and reorganization

This section includes four parts. The first part is the theoretical preparation where we define two important concepts: cSupB and offset value. The second part decomposes the graph and obtains all the cSupBs. The third part determines the relations between cSupBs, and the last part addresses cycles.

#### Colored superbubble (cSupB)

Before defining the cSupB, we need to know the definition of superbubble.

##### **Definition 1**

An assembly graph $G=(V, E)$ is a directed graph. Denote $V(G)$ and $E(G)$ as the set of nodes and edges, respectively, of graph $G$. For any two distinct nodes $s$ and $t$ in $G$, $<s, t>$ is called a **superbubble** if it satisfies the following four criteria [23, 39]:

1. reachability: there is a path from $s$ to $t$;
2. matching: the set of nodes reachable from $s$ without passing through $t$ is equal to the set of nodes from which $t$ is reachable without passing through $s$;
3. acyclicity: the subgraph induced by $U$ is acyclic, where $U$ is the set of nodes satisfying the matching criterion;
4. minimality: no node in $U$ other than $t$ forms a pair with $s$ that satisfies the conditions above;

Here, $s$ and $t$ are called the source node and sink node, respectively.

Based on Definition 1, there are two superbubbles in Fig. 1b:$<TAT,ACC >$; $<TCA,GTA>$. Here, we only discuss the superbubbles that contain at least two supernodes.

Many superbubble search methods have been developed [23, 39–41]. These methods can be applied to different graphs, and all are based on the topological structure. However, a superbubble contains structural information but lacks sample information; therefore, based on the colored de Bruijn graph, we propose the cSupB, a generalization of the superbubble, and its relevant algorithm.

##### **Definition 2**

An assembly graph $G = \left( {V,E,C} \right)$ is a colored de Bruijn graph. Denote $V\left( G \right)$, $E\left( G \right)$ and $C\left( G \right)$ as the sets of nodes, edges, and colors, respectively, of graph $G$. For a color set $C_{1}$ where $C_{1} \subset C$, $G_{1} = \left( {V_{1} ,E_{1} ,C_{1} } \right)$ is the subgraph induced by the nodes $u \in V$ whose color $color\left( u \right)$ satisfies $color\left( {u_{i} } \right) \cap C_{1} \ne \emptyset$. For any two distinct nodes $s$ and $t$ in $G$,$\left\langle {s,t,C_{1} } \right\rangle$ is called a cSupB if it satisfies the four criteria of a superbubble in graph $G_{1} .$

We can also define **separation, intersection, and inclusion** of cSupBs.

For any two different $cSupBs$ named $cSupB1$ and $cSupB2$, ${G}_{1}=\left({V}_{1},{E}_{1},{C}_{1}\right)$ and ${G}_{2}=({V}_{2},{E}_{2},{C}_{2})$ are the subgraphs induced by the nodes in $cSupB1$ and $cSupB2$ respectively. Then we set ${V}_{0}={V}_{1}\cap {V}_{2}, {C}_{0}={C}_{1} \cap {C}_{2}$ and we can determine the relations of $cSupBs$ according to five conditions as follows:

- (1) Separation: a. $${V}_{0}$$ = $$\varnothing$$; b. $${V}_{0}\ne \varnothing$$ and $${C}_{0}=\varnothing$$;
- (2) Intersection: c. ${V}_{0}\ne \varnothing$ and ${C}_{0}$ $\ne$ $\varnothing$ and ${V}_{0}\ne$ ${V}_{1}$ or ${V}_{2}$;
- (3) Inclusion: d. ${V}_{0}={V}_{1}$ and ${C}_{0}={C}_{1}$; e. ${V}_{0}={V}_{2}$ and ${C}_{0}= {C}_{2}$.

In particular, if two cSupBs are inclusive relations, for example: ${V}_{0}={V}_{2}$ and ${C}_{0}= {C}_{2}$, we name $cSupB1$ the parent bubble of $cSupB2 \mathrm{and }cSupB2$ is the child bubble of $cSupB1$.

Based on Definition 2, there are five cSupBs in Fig. 1b: bub1.$<TAT,ACC,111 >$; bub2.$<GGG,GTA,110>$; bub3.$<CAC,GTA,011>$; bub4.$<TCA,GGG,110>$; and bub5.$<TCA,GTA,111>$. Bub5 is the parent of bub2, bub3 and bub4. Here, we can prove that a superbubble is only a special group of cSupBs whose source and sink node colors are the same. Each cSupB can be thoroughly decomposed to simple cSupBs that have only two paths from the source node to the sink node. Simple cSupB is the minimum bubble structure that can be used to study relationships and variants for any two samples, but superbubbles cannot.

##### **Definition 3**

In a directed acyclic graph (DAG) $G=(V,E)$ with unique start node ${u}^{^{\prime}}$, for each node ${v}_{i}\in V$, let ${S}_{i}$ denote all the adjacent incoming nodes of ${v}_{i}$. If $pos({v}_{i})=max\{pos({u}_{j})\}+1$, where ${u}_{j}\in {S}_{i}$, and $pos({u}^{^{\prime}})$ is known, then $pos({v}_{i})$ is called the **offset value** of ${v}_{i}$.

Indeed, in a DAG $G=(V,E)$, for any two distinct nodes $u,v\in V$ and $pos\left(u\right)=a, pos(v)=b$, if there exists at least one path between $u$ and $v$, $|a-b|$ must be the length of the longest path between $u$ and $v$ (Additional file 1: property 3).

#### Graph traversal and cSupB Matching

Before traversing and decomposing the graph, we need to label four visiting states for eachnode as unvisited, half-visited, to-be-visited and fully visited. See Additional file 1 for the details of the description.

We define a **postorder-like traversal** strategy: in a DAG, **a node can be visited if all its parent nodes have been visited.** In other words, for each node in the graph, we could visit it and its outgoing nodes if and only if all its incoming nodes have been visited.

**Matching principle**: At least two adjacent outgoing edges’ colors of the source node intersect with the color of the sink node. Similarly, at least two adjacent incoming edges’ colors of the sink node intersect with the color of the source node. Then, the source node and sink node are matched, and the cSupB color is the color intersection of the source node and the sink node.

If we encounter a node $s$ whose outdegree is greater than one, we place it in a to-be-visited source node queue $Q$; if we encounter a node $t$ whose outdegree is greater than one, we start to find its matched source node $s$ from $Q$ reversely based on the matching principle. Specifically, if $color(s)\subseteq color(t)$, we remove $s$ from $Q$ and continue; if $color(s)\subseteq color(t)$, we stop.

We can prove that for each node $t$ whose indegree is greater than one, at least one node $s$ whose outdegree is greater than one can be used to construct a cSupB with $t$ (Theorem 2, Corollary 5). Moreover, $s$ is visited before $t$, and $s$ is the parent node of $t$ (Corollaries 3 and 4).

### cSupB subordination

After postorder-like traversal, we can obtain all the cSupBs and their information, including the source/sink node, cSupB color, and ordering. Ordering denotes the order of obtaining the cSupB, and we use the function $order$ denoting it in the following study. Here, we reorganized the cSupBs by determining the relationship of cSupBs.

To organize the cSupBs, we adopt a hierarchical tree structure by exploiting the relations of cSupBs. Furthermore, after confirming the root cSupB (containing all samples), we assigned each cSupB a hierarchical level. Here, the root cSupB’s level is 1.

### Method to determine the inclusion of cSupB

For $cSupB1$, $cSupB2$ and $cSupB3$, ${G}_{1}=\left({V}_{1},{E}_{1},{C}_{1}\right)$, ${G}_{2}=({V}_{2},{E}_{2},{C}_{2})$ and ${G}_{3}=({V}_{3},{E}_{3},{C}_{3})$ are the subgraphs induced by the nodes in $cSupB1,$ $cSupB2$ and $cSupB3$ respectively. If the following three conditions are satisfied simultaneously:

- $$\mathrm{a}.order({G}_{1}) < order({G}_{2})$$;
- ${\mathrm{b}.C}_{1}\subseteq {C}_{2}$ and ${C}_{1}\ne {C}_{0}$(${C}_{0}$ is a color set containing all samples);
- there is no $cSupB3$, s.t. $order\left({G}_{1}\right)< order\left({G}_{3}\right)< order({G}_{2})$ and ${C}_{1}\subset {C}_{3}$;

we call $cSupB1$ a child cSupB of $cSupB2$ and call $cSupB2$ the nearest parent cSupB of $cSupB1$ (Additional file 1: Theorem 3).

An essential conclusion is that a child cSupB cannot be obtained before its parent cSupB (Additional file 1: Corollary 6), and the cSupB structure must be a tree (Additional file 1: Corollary 7). Figure 1$D,E$ is an example of the final decomposition and representation of the colored de Bruijn graph.

To store various information about the colored de Bruijn graph, we define several file formats, such as cSupB topology (CST), cSupB detailed information (CSDI), node nearby information (NNI) and offset value information (OVI). See Additional file 1 for the details of the file formats.

#### Cycle

A cycle is the representation of repeat sequences on the graph and is sometimes common but inevitable. Cycles can be divided into two types: type I, formed by a single sample; and type II, formed by different samples. Our k value selection method can only avoid type I. Here, we propose a method to address cycles including whether cycles exist, where the cycles are and how to cut the sequences.

##### Cycle identification

There exists a depth-first-search strategy that can identify all the cycles simultaneously; however, the iterative recursion and high computational cost limit its application. In this paper, we propose a cycle identification method based on the offset value.

According to Theorem 2, if we traverse graph $G$ from the start node using the postorder-like traversal strategy, we can visit all the nodes. In particular, if the traversal is finished but nodes still exist that are unvisited, we call the traversal finished in advance and there must be cycles in graph $G$ (Theorem 2). Information about half-visited nodes is collected to prepare for cycle interval determination.

**Fig. 2:**
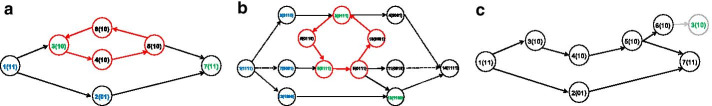
Half-visited nodes in two types of cycles. Each circle represents a node, and the red dots and edges form a cycle. In the circle, the number before the bracket indicates the node ID, and the number in the bracket represents the node color. The color of the number indicates the visiting state of the node: blue indicates fully visited, green indicates half-visited, and black indicates unvisited. **a** Graph composed of two samples, where the cycle belongs to type I. When the traversal stops, two half-visited nodes, 3 and 7, are generated. Among them, 3 is of the first type and 7 is of the second type. **b** Graph composed of four samples, where the cycle is a type II cycle. When the traversal stops, three half-visited nodes (3, 8, and 13) are generated. Among them, 3 and 8 are of the first type, and 13 is of the second type. **c** The revisiting result of **a**. When the traversal is finished in advance, two half-visited nodes (3 and 7) are obtained, and the intersection of the colors of the two nodes is not empty. At this time, $\mathrm{pos }(1) = 1,\mathrm{ pos }(3) =\mathrm{ pos }(2) = 2,\mathrm{ pos }(7) = 3$, and node 3 is selected as $\mathrm{cycle}\_\mathrm{ start}\_\mathrm{ node}$, $\mathrm{cycle}\_\mathrm{ start}\_\mathrm{ pos }= 2$. Then, by continuing to visit, we can obtain $\mathrm{pos }(4) = 3,\mathrm{ pos }(5) = 4,\mathrm{ pos }(6) =\mathrm{ pos }(7) = 5$ and know that $\mathrm{cycle}\_\mathrm{ end}\_\mathrm{ node}$ is 6, $\mathrm{cycle}\_\mathrm{ end}\_\mathrm{ pos }= 5$. Finally, the interval of the cycle is [2, 5]

We divide half-visited nodes into two types: type I: on the cycle; type II: not on the cycle. We can prove that if the traversal is finished in advance, we must obtain at least one type I half-visited node (property 5) (Fig. 2).

##### Cycle interval location

At least one half-visited node must exist on the cycle when the traversal ends in advance (property 5). Therefore, we need to identify this node, mark it as fully visited, and restart the graph traversal. Due to the existence of the cycle, this node is revisited, and we record the offset value information again to help us determine the interval of the cycle.

**Steps of interval determination**

- 1. Let ${V}_{0}$ represent the collection of all half-visited state nodes.
- 2. Choose node $u$ from ${V}_{0}$ whose offset value is minimal as the cycle start node.
- 3. Mark $u$ as fully visited and restart the graph traversal from $u$. If traversal is again finished in advance, we encounter a new cycle and return to step 1. If we meet a node $v$, $v$ is the adjacent parent node of $u$. Then, we set $cycle\_start\_pos= pos(u)$ $cycle\_end\_pos=pos(v)$, $cycle\_start\_node= u$, $cycle\_end\_node= v$.
- 4. Then, [$cycle\_start\_pos, cycle\_end\_pos$] is the cycle interval.
- We choose the node with the minimum offset value for simplicity. If the selected node is on the cycle, the traversal can proceed; otherwise, the traversal will terminate in advance, and the previous steps are repeated.

##### Sequence segmentation and graph reconstruction

After the interval [$cycle\_start\_pos, cycle\_end\_pos$] of the cycle is determined, we select a cutting point in this interval to divide the sequence into two parts and construct the graph.

Here, we divide the cutting points into three categories: type I: the bridge. This type is our ideal cutting point; type II: Not the bridge but involving all samples; type III: only involving a portion of the samples.

Based on the above ideas, we propose a cutting point selection model as follows.

1. Position transformation. We establish a connection between the offset value and bases. Here, we define the offset value of the node according to its last base in the k-mer.
2. Information obtainment. We take each position as the key and the color, bases, thickness, and other information as the values to establish hash tables in the interval [$cycle\_start\_pos, cycle\_end\_pos$].
3. Evaluation strategy. We set an index $R = L/t$, in which $L$ denotes the number of continuous loci with the same position information and $t$ denotes the thickness. In particular, if there are multiple choices with the same $R$-value, we prefer bridges; if the $L$ value is 0, only the position with the smallest thickness can be selected as the cutting position. In other cases, we make the decision according to the size of the $R$-value.
4. Cutting position determination. We select the optimal area as the reference cutting position. If there are multiple cycles, comprehensively considering the information of all cycles and their intersection should be the priority. The output format is cycle cut position (CCP) format (see Additional file 1 for details).

##### Sequence cutting and graph reconstruction

Although we know the cutting position of the graph, we must obtain the specific cutting position of each sample. We searched the cutting area sequence in each relevant sample. If there is only one search result, the middle position is the cutting position; if there is more than one search result, the nearest one is selected as the reference cutting position. Then, we cut each sequence, add the head and tail sequence again, divide them into several files, put them in multiple directories, and build graphs.

### Variant detection

Variant information of an individual or population can be deduced from the structure of the colored de Bruijn graph. Here, we propose a node-based variant detection algorithm.

In practice, the traversal starts from the end node of the graph and against the direction of the edge. The final offset value can be obtained using simple subtraction from the preoffset value shown in Fig. 1c.

We choose reverse traversal because the appearance of each source node means that a variant occurs. If it is a base substitution, the offset value is not affected; however, if it is an indel, using traversal along the edge direction, the offset break node is the sink node (node $GGG$ in Fig. 1b) instead of the source node (node $TCA$) we want. In this paper, we only discuss small indels (< 50 bp).

In the colored de Bruijn graph, we assume that the variant occurrence corresponds to the source node one-to-one, so we discuss variants near the source node. When traversing the graph, all the nodes' variant information is stored in the offset value; thus, the variant, source node, and offset value are closely linked.

**Fig. 3:**
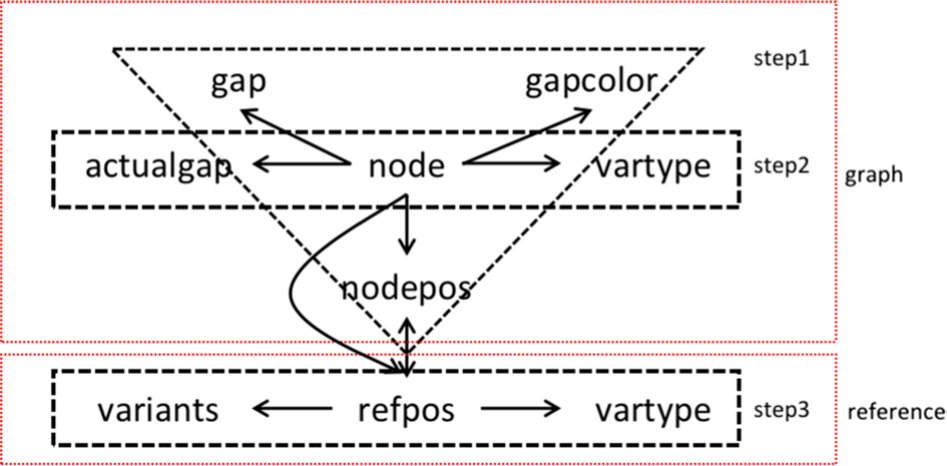
Variant identification and variable transformation

Here, we propose a **node-based** method:

1. Save the maximum gap length and color near each source node to obtain gap information;
2. Analyze the two types of source nodes (with reference and without reference) in turn, determine the variant type of each source node (nested variant detection algorithm (NVD)), and modify the gap at each source node to determine the node variant;
3. Determine the relations between the nodes' position on the reference genome and graph (reference position determination (RPD)); then, transform the node variant to a locus variant (Fig. 3).

### Nested variant detection (NVD) algorithm

#### Step 1: Detect variant types of source nodes involving the reference

**Fig. 4:**
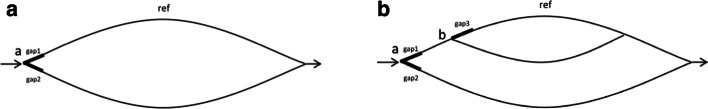
Different gap distributions near the source node

We traversed the cSupB id from small to large and only discuss the cSupB containing the reference genome. A simple situation is shown in Fig. 4a. If gap1 > 0, $var(a)=INS$; if gap2 > 0, $var(a)=DEL$; and if gap1 = gap2 = 0, $var(a)=SNP$. Here, function $var$ denotes the variant type of the node. For the more general case, for example, in Fig. 4b, if the variant types at nodes $a$ and $b$ are the single nucleotide polymorphism (SNP) and insertion (INS), respectively, the actual gap conditions are gap2 > 0 and gap3 > 0 based on the traversal against the direction of the edge. Therefore, since the correct results cannot be obtained by considering the gap of a node such as $a$ independently, we need to determine the node variant type more comprehensively. For detailed algorithm flows, see the NVD-1 flow chart in Additional file 1.

#### Step2: Detect variant types of other source nodes

This step traverses the cSupB id from small to large again and performs variant analysis on cSupB whose variant type has not been determined, that is, cSupB that does not contain the reference genome. The basic idea is to search the nearest parent source node containing the reference according to the cSupB tree structure when encountering a source node whose variant type has not yet been determined and record all the source nodes involved in turn. Then, by ordering from the parents to children, we can determine the variant types of all source nodes (see Additional file 1: father-children variant detection (FCVD)). For detailed algorithm flows, see the NVD-2 flow chart in Additional file 1.

##### Reference position determination (RPD)

All the source nodes can be divided into two types: source nodes contained in the reference genome or those that are not. For the former, we used a variable *refpos* to record its position in the reference genome. Therefore, the focus here is to infer the reference genome position of the second type of source nodes and finally obtain the approximate correspondence between the graph position *nodepos* and the *refpos*.

The basic idea here is similar to the NVD algorithm, and we still traverse the cSupB id from small to large. When encountering a source node in which the *refpos* has not been determined, we need to obtain its nearest parent source node that contains the reference according to the cSupB tree structure and record all the source nodes involved in turn. Then, following the ordering from parents to children, source nodes’ *refpos* can be determined, and the relationship between *refpos* and *nodepos* can also be obtained. Finally, we can simply obtain the variant at the reference genome site combined with the node variant information. For algorithm details, see the RPD flow chart in Additional file 1.

### Construction of the coordinate system

In the linear coordinate system, a simple positive integer $a$ and binary $(a, b)$ can be represented by the base and sequence information. The biological relationship between sequences can also be discussed. However, this representation is not suitable for genome graphs. Here, based on the cSupB tree model, we constructed a haplotype pangenome coordinate system.

We define a triple *(position, bubid, basecolor)* to represent each base location (BL) in a graph. Since every sample contained in any cSupB has only one path, this representation provides a one-to-one correspondence to nodes in the graph. The *position* represents the offset value of the bases, and *bubid* represents the smallest cSupB where the base is located. *basecolor* represents the samples that include this base at this position, which has two representations. One is to use a string consisting of ‘0’ and ‘1’, and its length is equal to the number of samples. This representation is same as the node or edge color. The other is to randomly select a sample id containing the base. The former is more comprehensive, and the latter is simpler: the selection can be made according to the application. In this triplet, *position*, *bubid* and *basecolor* provide numerical, topology and sample information, respectively.

Similar to the linear coordinate system, we discuss the sequence information. The sequence mapped to the graph is a path, which is represented by a six-tuple (path location (PL)), as follows:

$$\left( {startpos,startbub,endpos,endbub,pathbub,pathcolor} \right)$$

The *startpos* and *endpos* represent the offset values of the start and end nodes of the path, and *startbub* and *endbub* represent the smallest cSupB where the start and end nodes are located, respectively. *pathbub* represents the smallest cSupB that contains all the paths. *startbub* and *endbub* are both child bubbles of *pathbub*. If the path spans $n$ root cSupBs, *pathbub* is recorded as $-n$. The *pathcolor* represents the color of the path, which is represented as a 0–1 string. When the length of a path is equal to 1, the path is a base, *startpos* = *endpos*, *startbub* = *endbub* = *pathbub,* and *pathcolor* = *basecolor*. Then, the six-tuple of the path becomes a three-tuple of bases.

In the linear coordinate system, if two intervals are given, there are three kinds of relations: separation, intersection, and inclusion. Similarly, on the genome graph, we can provide the correlation between two paths.

Given two random paths, path1: *(a1, bub1, b1, bub2, bub3, color1)* and path2: *(a2, bub4, b2, bub5, bub6, color2)*.

1. If there is no intersection between [*a1, b1*] and [*a2, b2*], path1 and path2 are separated;
2. If the relationship between [*a1, b1*] and [*a2, b2*] is inclusion and *color1* and *color2* also have an inclusion relationship, then path1 and path2 have an inclusion relationship;
3. In other cases, path1 and path2 intersect.

Specifically, if [*a1, b1*], [*a2, b2*] have an intersection and *color1* and *color2* do not intersect, then path1 and path2 intersect. At this time, sequence similarity analysis can be performed. According to the cSupB tree, we can find the nearest parent bubble *bub7* of *bub3* and *bub6* and its color *color3*. If *bub7* does not exist, the path spans the root cSupB, and *color3* includes all samples; if *bub7* exists, similarity analysis can be performed in *bub7*, and the union of *color1* and *color2* is a subset of *color3*.

Taking Fig. 1c as an example, we know that there are five cSupBs bub1.$<TAT,ACC,111 >$; bub2.$<GGG,GTA,110>$; bub3.$<CAC,GTA,011>$; bub4.$<TCA,GGG,110>$; and bub5.$<TCA,GTA,111>$. We randomly select a node such as TCA, and its base position is (3, 4, 111) in which 3, 4, and 111 denote TCA’s offset value, smallest cSupB id and base color, respectively. Similarly, we can also obtain other nodes’ base positions such as ATG: (13, 1, 100), CCC: (17, -1, 111) and GGG: (7, 2, 110). Then, we randomly select three paths: $a$. CAGGGTGTA- > (5, 4, 11, 2, 5, 100); $b$. GGGAGTA—> (7, 2, 11, 2, 2, 010); and $c$. ATAACCC- > (13, 1, 17, 1, 1, 011). Taking path $a$ as an example, we give a brief introduction to its components. The first node of path $a$ is CAG, whose base position is (5, 4, 100), and the last node GTA’s base position is (11, 2, 111). All the nodes on path $a$ are contained in cSupB5, and their intersection of node colors is 100. Here, path $a$ and path $b$ intersect because they have common nodes such as GGG, and path $a$($b$) and path $c$ are separated because there is no intersection between [5, 11] ([7, 11]) and [13, 17].

Based on the genome graph coordinate system and variant detection algorithm, we can achieve more functions. For example, given a genome annotation file (e.g., gene transfer format (gtf), general feature format (gff)), we can obtain the relations between the annotation information and the topological structure and predict all the variants; if given a variant file (e.g., variant call format (vcf)), we can not only find the topological information based on the variants but also predict the variant information and compare it to the variant file to explore further discoveries.

## Results

We evaluated the method performance on four different datasets described below. For this evaluation, we discussed the performance of graph decomposition, the mapping between graph and variant (annotation) files, variant detection, and cycle processing with dataset 1. To further evaluate the variant detection method, we compared precision and recall rate with other tools with dataset 2. In addition, we also assessed the computational ability for large-scale genomes with simulated dataset 3 and dataset 4.

### Datasets

The human mitochondrial genome has the following advantages: (1) the high variant rate ensures that a large number of variants can be used; (2) the rich genome data contains almost all common and abnormal situations, and we can select the required data according to the concrete purpose; (3) the quality of the mitochondrial genome is high, which is consistent with our assumption that the genome represents the true genetic information of the individual. In addition, other research results, such as annotations, may help verify the accuracy of the model.

Four datasets were chosen to test and evaluate the performance of *cSupB*. Dataset 1: 12 human mitochondrial genomes, including a reference genome [42]. Sample names are AP008459, AP010675, EF153784, EF153791, EF153794, EF153814, EF397559, EU007868, FJ493500, GQ895144, GU377085, and NC_012920. Dataset 2: A series of random simulations with 10 samples for each time. Dataset 3: 5000 randomly simulated human mitochondrial genomes. Dataset 4: 20,000 randomly simulated human mitochondrial genomes. The simulation method is provided in Additional file 1.

### Implementation of the tri-tuple coordinate system

Given 12 human mitochondrial genomes (see Additional file 2), we calculated that the longest repeat length of the samples was 16, except that of AP010675, which was 20, and its repeat region was TATAGCACCCCCTCTACCCCCTCTACCCCCTCTA, in which two repeats intersected. When the k value is greater than 20, the constructed colored de Bruijn graph is a DAG; otherwise, the constructed graph is a directed cyclic graph. Here, we set k = 22 for VARI-merge to construct an acyclic graph. In addition, there was a degenerate base N at the 3107th position in the reference genome, and we replaced N with C to avoid affecting the annotation information.

Using the postorder-like traversal strategy, we traversed the colored de Bruijn graph only once to find all cSupBs and their affiliation. In total, 118 cSupBs were identified, including 74 root cSupBs (65 simple cSupBs) and the largest cSupB tree, whose level was 5. These results are shown in Table 1, and more information can be obtained from the CST file.

**Table 1:** Comparison of the graph decomposition results of two k values

k	Graph	All cSupBs	Root CSupB	Max level	Simple cSupB
22	Graph	118 (74 + 20 + 14 + 9 + 1)	74	5	91 (65 + 26)
18	Graph1	55 (37 + 8 + 6 + 4)	37	4	44 (33 + 11)
	Graph2	64 (37 + 15 + 7 + 4 + 1)	37	5	47 (32 + 15)

After obtaining the necessary information about cSupBs, we can collect more detailed information about each cSupB stored in the CSDI file. This additional information includes the node/edge/branch/supernode count, sample length, and branch information.

We can also map the existing annotation file to the graph: 578 of 3892 variants in the mtDNA vcf file are located in cSupB regions, and the others are located on bridges. Additionally, 37 genes in the gtf file are located on the bridges or span at least one cSupB, as shown in Table 2. All the variants and gene coordinates can be seen in vcf2graph.txt (see Additional file 3) and gtf2graph.txt (see Additional file 4).

**Table 2:** The statistics of cSupBs involved in gene regions

cSupB included	0	1	2	3	4	5	6	8	
Count	21	5	1	3	1	3	2	1	37

While obtaining the cSupB relations, we detected the variants using the node-based variant detection algorithm. The statistical results of the variants are shown in Tables 3 and 4. Here, vartype indicates different variant types (1: substitution, 2: deletion, 3: insertion, 4: indel (unsure 2 or 3)).

**Table 3:** Node variant statistics

Vartype	1	2	3	4	
Count	82	3	6	1	92

**Table 4:** SNV statistics

Vartype	1	2	3	4	
Count	82	9	17	2	110

Compared to the mtDNA annotation file downloaded from 1000 Genomes (ftp://ftp.1000genomes.ebi.ac.uk/vol1/ftp/release/20130502/ALL.chrMT.phase3_callmom-v0_4.20130502.genotypes.vcf.gz), 89 of 110 single nucleotide variants (SNVs) can be location mapped. Moreover, 20 of 21 unmapped images can be verified for accuracy, as shown in Table 5.

**Table 5:** SNVs that cannot be found in the annotation files but that actually exist

Position	Ref	Alt	Vartype	Involved sample
97 ~ 102	GCTGGA	-	Deletion	EF153794
449	T	C	SNP	EF397559
632	C	T	SNP	GQ895144
3213	A	G	SNP	EF153794
4658	A	G	SNP	EF153784
6101	C	T	SNP	EF153794
8290–8299	G	CCCCCTCTAG	Insertion	AP010675
13,086	C	T	SNP	EF153784
15,142	C	T	SNP	FJ493500
15,814	A	G	SNP	EF153784

The overall flow includes graph construction, decomposition, reorganization, and variant determination, and the total calculation time is less than 1 min. To further assess the computational ability for large-scale genome data, we simulated 5000 and 20,000 mitochondrial genomes. The calculation time shown in Table 6 is divided into two components: graph construction and analysis. Our algorithm is minimally constrained by the sample size; allowing for constructing the graph model we need, we can quickly obtain the decomposition result.

**Table 6:** Calculation time for two large-scale datasets

Samples	5000	20,000
*cSupB*	7.5 h + 3 min	30.5 h + 8 min

### Assessment of the variant detection accuracy

Two factors are related to the node-based variant detection algorithm: the number of variants, such as SNP, DEL, and INS, and the k value. Therefore, we used simulated data based on the different parameters mentioned above to evaluate the model. To assess the variant detection results, we used multiple sequence alignment tools, including multiple sequence comparison by log-expectation (MUSCLE) [43], multiple sequence alignment based on fast Fourier transform – GINSI alignment (MAFFT-GINSI) [44], Clustal Omega [45], and Mugsy [46], to identify SNVs, calculate the precision and recall rate, and compare the results. All these tools use a single CPU thread.

**Fig. 5:**
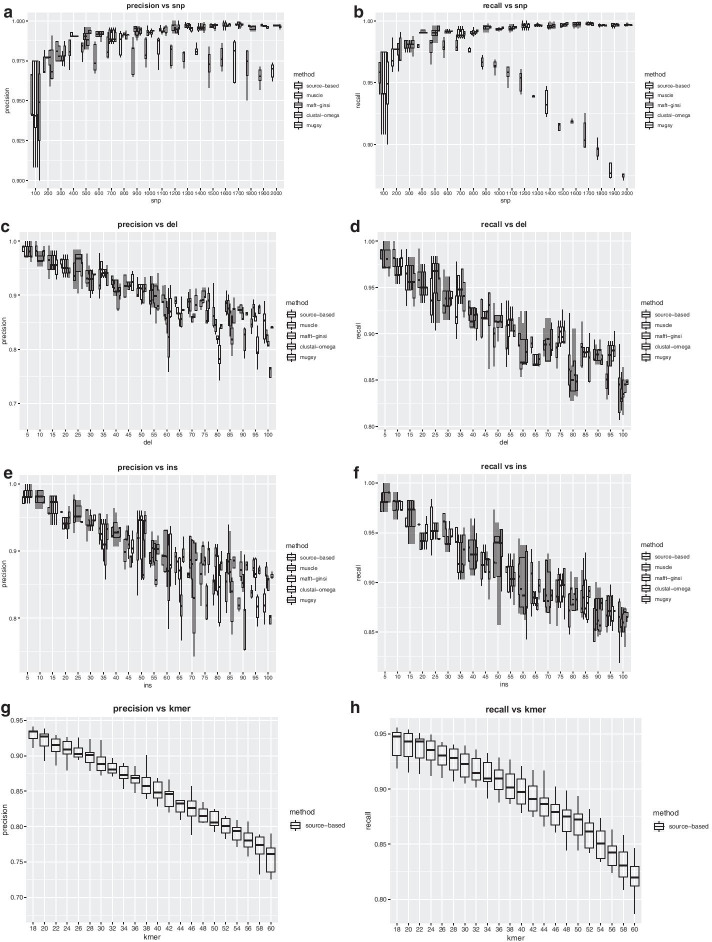
Precision and recall for different simulation parameters

Our simulation can be divided into four parts based on the experimental parameters, and the detailed parameters are listed in Table 7. For each set, we simulated 10 human mitochondrial genomes, including a reference genome. Here, precision and recall are calculated by means of location-mapped variants (Fig. 5), and the results used by type-mapped variants are shown in Additional file 1.

**Table 7:** Parameters of the simulation

Part	Experimental parameter	Para1:SNP	Para2: deletion	Para3: Insertion	Para4: k value	Set (groups*repeats)
1	SNP	100–2000 (0.6–12.0%)	10(~ 0.06%)	10(~ 0.06%)	28	20*5
2	Deletion	100(~ 0.60%)	5–100 (0.03–0.60%)	0	24	20*5
3	Insertion	100 (~ 0.60%)	0	5–100 (0.03–0.60%)	24	20*5
4	k value	500(~ 3.0%)	50(~ 0.3%)	50(~ 0.3%)	20–60	22*10

The node-based variant detection algorithm has high accuracy. For each simulation and each parameter, there is little difference between the various methods. In some sets, our method has the best precision and recall rate. In addition, our method has the best stability.

Indel has a greater impact than SNP. Additionally, Fig. 5a, b show that as the number of SNPs increases, the precision and recall increase gradually. However, simulations of deletion and insertion show the opposite trend, and the indel variable has a significant impact on the results (Fig. 5c, d, e, f). Theoretically, if there are only SNP variants, the precision of our method is 100%.

The k value is closely related to the structure of the graph: as the k value increases, the precision and recall decrease continuously (Fig. 5g, h). The reason is that when the k value increases, the k-mer becomes longer, the opportunity for node merging increases, and the structure of the graph becomes loose, which leads to a reduction in the source node number. Therefore, regardless of other factors, when we select the k value, the smaller the k value is, the better.

Furthermore, as the variants continue, the precision and the recall decrease. We know that indel variants have a great negative effect on precision and recall. In a certain range, the precision and recall increase with SNV variants, but the recall decreases sharply when the SNV mutation rate is outside of this range. The main reason is that the variant results cannot be mapped to the graph due to the decrease in sequence similarity; that is, the source number is far less than the variant number, which is not in line with our primary hypothesis of direct variant and source one-to-one correspondence. Thus, if the sequence similarity is insufficient, the variant detection model is not applicable.

In short, the higher the sample similarity is, the higher the precision and recall of SNVs for the node-based variant detection method.

### Cycle processing

To test the reliability of cycle processing, we used dataset 1 as an example and set k = 18. After analyzing the graph, we found two cycles, as shown in Table 8.

**Table 8:** Basic information about the two cycles

Id	cycle_start_node	cycle_end_node	cycle_start_pos	cycle_end_pos	Half-visited nodes	Type
1	199	71	405	16,288	5	II
2	8532	11,846	8282	8290	1	I

Selection of the cutting point.

**Fig. 6:**
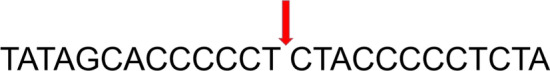
Final cutting region and reference cut position

- Combine adjacent cutting points of the same type and calculate the $R$-value. Here, cycle 1 has a total of 145 integrated cutting areas, in which the numbers of the three types of points are 73, 66, and 6. The $R$-value ranges from 0.5 to 1236; cycle 2 has only one type I cutting area, and the length is 26.
- Cutting point selection. Here, cycle 2 is included in cycle 1, and the cutting area of cycle 2 is part of cycle 1. The $R$-value of cycle 1 in this cutting area is 1236, which is the most ideal cutting area. Therefore, we chose only one cutting area of cycle 2 as the final cutting area, output the base information and position information of the cutting area as the reference position, and select the middle position as the reference cutting point, as shown in Fig. 6.

### Sequence cutting and graph reconstruction

Next, the segment of the cutting region is searched in the original sequence file. Then, we placed the sequences in the same area into the same directory. For each colored de Bruijn graph we constructed, we ensured that all sequences had the same head and tail. Therefore, before reconstructing the colored de Bruijn graph from the cutting sequences, we need to supply the same head and tail; that is, if the head/tail is missing, we added the appropriate head/tail. After the sequence preparation is complete, the previous construction steps are followed to rebuild the colored de Bruijn graph.

The statistical results are shown in Table 1. Notably, sequence cutting does not substantially impact the topological structure of a graph.

## Discussion

We present *cSupB*, which is an implementation of graph decomposition and reorganization, variant detection, and coordinate system design of the colored de Bruijn graph. First, we constructed a set of population haplotype genomes into a DAG with a unique start and end node. On the basis of the graph, we proposed a cSupB data structure that incorporates sample information, such as node sources and path linkages, while inheriting the features of superbubbles. Then, we can quickly obtain cSupB structure trees through traversing, matching and relationship determination. Moreover, to describe the node location information, we introduced the offset value to measure the length of the longest path from the start node to each node. Ultimately, we proposed a node-based algorithm to identify variants in specified samples and construct a genome graph tri-tuple coordinate system.

Despite the relative effectiveness of the reference as a coordinate system, using the reference as a lens to study all other genomes introduces a pervasive reference bias, especially in the population genome era. Our coordinate system, which satisfies monotonicity, readability, and spatiality, provides a means to discuss the genome graph in terms of topologic structure to genetic element location. Indeed, the number of sequences is amenable to a global or local directed acyclic representation, and our algorithm is compatible with any directed acyclic colored graph; thus, it has wide application prospects. Additionally, we can discuss the relationships of any paths or samples quickly without reconstructing and querying once the acyclic graph is constructed. The coordinate system can also handle repetitive events. By selecting appropriate k values or cutting sequences, we can transform genomes into one or more directed acyclic colored de Bruijn graphs. However, the method of sequence cutting has certain limitations; that is, for highly repetitive sequences, especially tandem repeats, the cutting method will make the sequence too fragmented, thereby increasing the computational difficulty and error.

The essential assumption of the node-based variant detection algorithm is that the generation of the variant corresponds to the source node in a one-to-one manner. Only the gap information and the cSupB structure tree near the source node are needed to obtain the final variant information efficiently. We demonstrated that when the similarity of the sample decreases, the variant type becomes complicated, and the precision of the algorithm is reduced. Therefore, this approach is applicable only when the sample has a high similarity and the variant type is relatively simple. In addition, the choice of the k value impacts the accuracy of the algorithm, and we preferred a smaller k value to ensure acyclicity.

With the introduction of more chromosome-level and haplotype-resolved assemblies, our decomposition and reorganization method could also be effectively handled. Moreover, this method is not limited to the VARI/VARI-merge tool, even the colored de Bruijn graph, but can theoretically be generated for any colored DAG. Compared to other motifs, such as ultrabubbles, the cSupB we proposed overcomes the limitation of topology but cannot handle more complex situations. Therefore, truly universal and comprehensive spatial framework research for genomic graphs will become a focus in the future. We anticipate that these studies will allow graph research to become a more practical analysis approach and increase the accessibility of existing possibilities.

Nevertheless, our model has several shortcomings. First, although graph research has steadily improved in recent years, almost all colored de Bruijn graph construction tools, including the VARI-merge used in this paper, do not have functions such as mapping, which significantly limits data selection and model promotion. Second, if the length of the gene element is less than k, there may be multiple corresponding nodes on the graph, which makes the analysis complicated and ambiguous. Third, we cannot handle degeneracy efficiently. Fourth, our variant detection method could only address small indels, but for more complicated conditions, such as large indels, inversions, and reverse tandem duplications, we do not have an effective solution; thus, we expect to introduce more high-quality genomes and comprehensive tools to perform genome graph research in the future.

In short, we anticipate that the genome graph research model presented in this paper will not only be compatible with more abundant information but also introduce additional computational methods to explore potential biological functions. We believe strongly that with the arrival of the population genome era, genome graphs and their spatial structure will be deeply studied and will play an important role in the research on population genomic diversity.

## Supplementary information

**Additional file 1** Proofs, file format details, variant detection method, simulation method and additional variant accuracy calculation results.

**Additional file 2** Twelve human mitogenomes used in the article.

**Additional file 3** The variant position coordinates on the graph in the mitochondrial genomevariant call format (vcf) file

**Additional file 4** The gene region coordinates on the graph in the mitochondrial genome genetransfer format (gtf) file.

## Data Availability

The datasets analyzed and the C +  + program implemented during the current study are available at https://github.com/eggleader/cSupB.

## Abbreviations

BL: Base location
cSupB: Colored superbubble
CCP: Cycle cut position
CSDI: CSupB detailed information
CST: CSupB topology
DAG: Directed acyclic graph
NNI: Node nearby information
NVD: Nested variant detection
SNP: Single nucleotide polymorphism
SNV: Single nucleotide variants
OVI: Offset value information
PL: Path location
RPD: Reference position determination
