# Supplementary material for: A tri-tuple coordinate system derived for fast and accurate analysis of the colored de Bruijn graph-based pangenomes
Source: BMC Bioinformatics. 2021 May 27;22:282. doi: 10.1186/s12859-021-04149-w (PMC8161984; doi:10.1186/s12859-021-04149-w)
Supplement: Supplementary file 1 — Additional file 1 Proofs, file format details, variant detection method, simulation method and additional variant accuracy calculation results. [file 12859_2021_4149_MOESM1_ESM.docx]

**Supplementary materials of “****A tri-tuple coordinate system derived for fast and accurate analysis of the colored de Bruijn graph-based pangenomes”**

**Content**

[1. Proof of properties, theorems, and corollaries 1](#_Toc68964444)

[2. Traversal and matching 6](#_Toc68964445)

[3. Output file format 10](#_Toc68964446)

[4. Variant detection method 12](#_Toc68964447)

[5 Simulation method 17](#_Toc68964448)

[6 Calculation result 18](#_Toc68964449)

# 1. Proof of properties, theorems, and corollaries

In a directed acyclic colored de Bruijn graph $G=(V,E,C)$ with unique start and end nodes $u^{'},v^{'}$, traversing by postorder-like strategy start from the start node, we can draw the following conclusions:

**Property 1**: Each sequence has only one path on the graph that corresponds to it.

Proof: Existence. Every time moving one stride, we can obtain a new k-mer which is adjacent to the previous one. Each k-mer corresponds to a node and the continuity of the sequence ensures the continuity of the nodes, so there must be a path corresponding to the sequence.

Uniqueness. By contradiction, if the path is not unique, there must be a divergence like the structure of fig. 1A or 1B in the graph. Here, taking fig. 1A as an example, there are two paths starting from $u1$ ($u1\to u2$, $u1\to u3$) in the graph. Supposing $u2$ appear ahead of $u3$ in the sequence, so there exists a path from $u2$ to $u3$. Then there must exist a path $u1,u2,\ldots,u1,u3$ containing a cycle structure which contradicts with the acyclicity, so the structure of fig. 1a does not exist.

Similarly, the structure of fig. 1B does not exist either. Therefore, in the directed acyclic colored de Bruijn graph, there is only one path corresponding for each sequence.


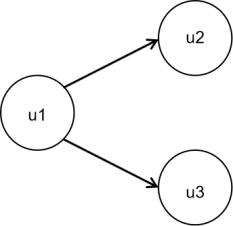

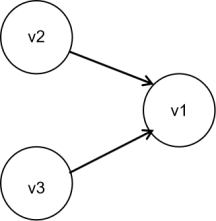


A B

Fig. 1 The possible structure if a sequence corresponds to mulple paths

**Property 2**: For any cSupB, $n$, $e$, $b$, and $s$ are the counts of node/edge/branch/ supernode, then $e+s=n+b$ is satisfied.

Proof: In a cSupB, supernode represents nodes with in-degree or out-degree greater than one. If all the supernodes are removed, the remaining cSupBs are disconnected branch-like structures, where the number of edges is one more than the nodes. There are $b$ such structures, that is, the number of edges in cSupB is greater by $b$ than the nodes after removing the supernodes, so $e-(n-s)=b$, and $e+s=n+b$ after shifting the term.

**Property 3**: For any nodes $u,v\in V$, if there is at least one path between them, and set $pos(u)=a，pos(v)=b$, then $|a-b|$ is the must be the length of longest path between $u$ and $v$.

Proof: By contradiction, suppose that there is a path $P$ from $u$ to $v$ satisfying $dist\left( u,v \right)>b-a=pos\left( v \right)-pos(u)$. Starting from $u$ along the $P$, there is at least one node $w$ (if there is only one, $w=v$) and let $w$ be the first node such that $pos\left( w \right)-pos\left( u \right)<l$, $l$ represents the distance from $u$ to $w$ on $P$. If the in-degree of $w$ is 1, it is contradictory to the first conflicting node of $w$; if the in-degree of $w$ is greater than 1, setting $\{w_{j}\}$ denotes all the adjacent incoming nodes of $w$ and let $w_{1}$ denote the incoming nodes on $P$ satisfying $pos\left( w_{1} \right)=pos\left( u \right)+l-1$, at this time $pos\left( w_{j} \right)\leq pos\left( u \right)+l-1$, otherwise it is contradictory with $P$ being the longest path. According to the definition, $pos\left( w \right)=pos\left( u \right)+l$ contradicts with $pos\left( w \right)-pos\left( u \right)<l$. Therefore, the original proposition holds.

**Theorem 1**: (Ordering) Let $u$ denote any node marked as visited, then all the parent nodes of $u$ must have been visited.

Proof: By contradiction, suppose there is a parent node $v$ of $u$ that has not been visited while $u$ has been visited. Because $v$ is the parent node of $u$, there must be a path $P$ from $v$ to $u$. According to our postorder-like traversal strategy, a node and its child nodes can be visited if only when all the incoming nodes of it have been visited. Then the adjacent node $w$ to $u$ on the path $P$ must have been visited, and so on, $v$ must have been also visited, which contradicts with the assumption. Therefore, for any node that has been visited, all parent nodes of it must also be visited.

**Corollary 1**: Let $S$ denote the samples contained in any node $u$ that has been visited, then the corresponding nodes for all sequences of $S$ on the 3' side of $u$ have also been visited.

Proof: In the directed acyclic colored de Bruijn graph we defined, each sample has only one path in colored de Bruijn graph. If $s_{1}$ is a sample contained in $u$ that has been visited, then the nodes corresponding to the sequence of $s_{1}$ on the 3' side of $u$ are the parent nodes of $u$. According to property 1, since node $u$ has been visited, it can be known that these parent nodes must also be visited.

**Theorem 2**: (Full coverage) All nodes must be visited. In particular, if the traversal is finished but still existing nodes unvisited, there must be a cycle in the graph.

Proof: By contradiction, if there is a node $v_{0}$ that has not been visited, it means that this node is half-visited or unvisited. Then when visiting from the opposite direction of $v_{0}$, there must be at least one half-visited or unvisited incoming node $v_{1}$ and so on. Then a path $\cdots v_{k}\cdots v_{1}v_{0}$ can be obtained, denoted as $P$, where the state of each node on $P$ is half-visited or unvisited. Because of the finite number of nodes, there are two traceability results for $P$. One is to trace back to a node with an indegree of 0 and then the traversal stops, but there is only one node whose indegree is 0, which is the start node. This case is not possible because start node must have been visited; the second is that there are two nodes $v_{i}$,$v_{j}\in P$, satisfying $v_{i}=v_{j}$, that is $v_{i}v_{i+1}\cdots v_{j-1}v_{j}$, which is a cycle. This contradicts with the acyclicity of the graph. So, all nodes can be visited.

In particular, if the traversal is finished in advance, indicating that the end node has not been visited, then let $v_{0}$ equal to the end node and repeat the above operations to prove the existence of circles.

**Corollary 2**: We can visit all nodes in turns to reach $v^{'}$ by the postorder-like traversal strategy start from $u^{'}$.

Proof: The corollary here has two meanings, ordering and full coverage. Ordering means that the child nodes will not be visited before their parent nodes. Full coverage means that all nodes can be visited, without omission. These two properties can be proved separately by the ordering and full coverage theorems, so the corollary holds.

**Corollary 3**: The sink node must not be visited before its source node.

Proof: For any pair of the source node ($s$) and sink node ($t$), according to the matching conditions, there are at least two paths from $s$ to $t$, then $s$ must be the parent node of $t$. According to Theorem 1, when $t$ is visited, $s$ must have been visited and the visit time is earlier than t.

**Corollary 4**: (Consistency) For any two nodes $u, v$, if $order(u)<order(v)$ and $color(u)\cap color(v)\neq\emptyset$, then $u$ must be the parent node of $v$.

Proof: By contradiction, $u$ is not the parent node of $v$. Since the color intersection of $u, v$ is not empty, it means that $u$ and $v$ exist in at least one sample in common. Also, each sample has only one path in the graph so $u$ and $v$ must be each other's parent and child node. If $u$ is not the parent node of $v$, then $u$ must be the child node of $v$. Because $order(u)<order(v)$, we go back to the previous step of visiting $v$. At this time, $u$ is visited but $v$ is not visited, that is, the situation that the child node is visited but the parent node is not visited contradicts with the theorem 1, so hypothesis does not hold. Therefore, $u$ must be the parent node of $v$.

**Theorem 3**: (Existence) For any node $u$ and a sink node $v$, if $order\left( u \right)<order\left( v \right)$, $C_{1}=color\left( u \right)\supseteq color\left( v \right)=C_{2}$, then during the traversal from $u$ to $v$, there must be a node $w$ so that $<w,v,C_{2}>$ exists. Similarly, for any source node $u$and a node $v$, if $order\left( u \right)<order\left( v \right)$, $C_{1}\subseteq C_{2}$, then there must be a node $w$ in the traversal from $u$ to $v$ such that $<u,w,C_{1}>$ exists.

Proof: In the directed acyclic colored de Bruijn graph, each sample has only one path in the graph. For any node $u$ and a sink node $v$, if $order\left( u \right)<order\left( v \right)$ and $C_{1}\supseteq C_{2}$, it can be seen from Corollary 4 that $u$ is the parent node of $v$, and all paths backtracking from $v$ are certain through $u$. Since $v$ is a sink node, that is, $v$ has at least two adjacent incoming nodes, and we take these nodes as the start to backtracking in the graph, satisfying that the node $u_{0}$ visited during the backtracking satisfies$color(u_{0})\cap C_{2}\neq\emptyset$. In the process of tracing back to $u$, the first node $w$ (including $u$) which satisfies $color\left( w \right)=C_{0}\supseteq C_{2}$ must be encountered, and $w$ must be a source node that satisfies any of the adjacent outgoing node $w_{i}$, $\emptyset\neq color(w_{i})\cap C_{2}\subseteq C_{2}$. At this time $<w,v,C_{2}>$ is a cSupB.

Similarly, for any source node $u$ and a node $v$, if $order\left( u \right)<order\left( v \right)$, $C_{1}\subseteq C_{2}$, then there must be a node $w$ in the traversal from $u$ to $v$ such that $<u,w,C_{1}>$exists. Except for the forward traversal from $u$, the other proof process is the same as above.

**Corollary 5**: For every node whose outdegree is greater than 1, there exists at least one cSupB taking it as the source node in the colored de Bruijn graph, and similarly, for each node whose indegree is greater than 1, there is at least a cSupB whose sink node is it.

Proof: Due to the existence of source node $s_{0}$and sink node $t_{0}$ containing all samples, any node belongs to a cSupB formed by the nearest $s_{0}$ and $t_{0}$. The colors of all nodes in this cSupB are all derived from $s_{0}$ and converged to $t_{0}$. Any source node $u$ in this cSupB satisfies $order\left( u \right)<order(t_{0})$ and $color\left( u \right)\subseteq color\left( t_{0} \right)$. By theorem 3, we can see that from $u$ to $t_{0}$, there must be a node $w$, so that $<u,w,color(u)>$ exists. In the same way, it can be proved that the latter is also true.

**Corollary 6**: The child cSupB will not be obtained before its parent cSupB.

Proof: If $cSupB1$: $<s_{1},t_{1},C_{1}>$ is a child cSupB of $cSupB2$:$<s_{2},t_{2},C_{2}>$ and $G_{1}=\left( V_{1},E_{1},C_{1} \right)$and $G_{2}=(V_{2},E_{2},C_{2})$ are the subgraphs induced by the nodes in $cSupB1$ and $cSupB2$ respectively, it means that $C_{1}\subseteq C_{2}$, and $V_{1}\subset V_{2}$. From the uniqueness of the path for each sample in the graph, $s_{2}$ is the parent node of $s_{1}$. According to theorem 1, $order(s_{2})\leq order(s_{1})$. In the same way, we can see that $order(t_{1})\leq order(t_{2})$, but two equal signs cannot be satisfied simultaneously, otherwise two cSupBs are the same. If $order\left( s_{2} \right)=order(s_{1})$, then $order\left( t_{1} \right)<order(t_{2})$, obviously $order(G_{1}) < order(G_{2})$; if $order\left( t_{1} \right)=order(t_{2})$, then $order\left( s_{2} \right)<order(s_{1})$, when traversing the source queue, first judging whether $s_{1}$ meets the matching condition and then judging $s_{2}$, thus $order(G_{1}) < order(G_{2})$ also holds.

**Lemma 1**: For any three cSupBs, $G_{1}=\left( V_{1},E_{1},C_{1} \right)$, $G_{2}=(V_{2},E_{2},C_{2})$ and $G_{3}=(V_{3},E_{3},C_{3})$ are the subgraphs induced by the nodes in $cSupB1:<s_{1},t_{1},C_{1}>,$ $cSupB2$:$<s_{2},t_{2},C_{2}>$ and $cSupB3:<s_{3},t_{3},C_{3}>$ respectively, satisfying $order(G_{1}) < order(G_{2})$and $C_{1}\subseteq C_{2}$, and there is no other cSupB3 satisfying $order(G_{1}) < order(G_{3}) < order(G_{2})$ and $C_{1}\subseteq C_{3}$, then the order of the nodes satisfies $order\left( s_{2} \right)\leq order\left( s_{1} \right)<order(t_{1})\leq order(t_{2})$ and the equal signs are not satisfied simultaneously.

Proof: Since $order(G_{1}) < order(G_{2})$ and $order\left( s_{1} \right)<order\left( t_{1} \right)\leq order(t_{2})$, we only need to prove $order\left( s_{2} \right)\leq order\left( s_{1} \right)$.

If $order\left( s_{1} \right)<order\left( s_{2} \right)<order(t_{1})$, due to $C_{1}\subseteq C_{2}$, then $C_{1}\cap color\left( t_{2} \right)\subseteq color\left( t_{1} \right)\cap C_{1}$ is satisfied. It can be known from theorem 3 that in the traversal from $t_{1}$ to $t_{2}$, there must be a node $s_{2}^{’}$, so that cSupB3: $<{s_{2}^{’},t}_{2},C_{1}>$ exists, at this time, $order\left( G_{1} \right)< order(G_{3}) < order(G_{2})$ and $C_{1}\subseteq C_{1}$.

If $order\left( t_{1} \right)\leq order\left( s_{2} \right)<order(t_{2})$, there must be a sink node $t_{3}$ whose color contains $C_{2}$ due to $C_{1}\subseteq C_{2}$ from $t_{1}$ to $s_{2}$. Then from the nearest parent root source node $s_{0}$ to $t_{3}$, by theorem 3, we know that there must be a source node $s_{3}$ such that cSupB3: $<s_{3}{,t}_{3},C_{2}>$ holds, then $order(G_{1}) < order(G_{3}) < order(G_{2})$ and $C_{1}\subseteq C_{2}$ are satisfied, too.

So, $order\left( s_{2} \right)\leq order\left( s_{1} \right)<order(t_{1})\leq order(t_{2})$ is satisfied and the equal signs are not satisfied simultaneously otherwise cSupB1 and cSupB2 are the same.

**Theorem 4**: The cSupB obtained according to the three conditions of cSupB inclusion relationship must also satisfy the inclusion relationship of the nodes.

Proof: According to Lemma 1, for any two cSupB, cSupB1: $<s_{1},t_{1},C_{1}>$ and cSupB2: $<s_{2},t_{2},C_{2}>$, if the three conditions for determining the inclusion relationship are satisfied , then the nodes visiting order satisfies $order\left( s_{2} \right)\leq order\left( s_{1} \right)<order(t_{1})\leq order(t_{2})$ and the equal sign does not hold at the same time. From Corollary 4, we can see that there are two nodes whose colors have an intersection, and the first visited node must be the parent node of another and the color satisfies $C_{1}\subseteq C_{2}$. Based on the uniqueness of each color path, all paths that can be visited from $s_{2}$ to $t_{2}$ must include the paths from $s_{1}$ to $t_{1}$, so cSupB2 contains all the nodes in cSupB1.

**Corollary 7**: The topological structure determined by the cSupB inclusion relationship is a tree.

Proof: According to the method of determining the inclusion relationship of cSupBs, we can know that there is a minimal ordering distance between the child cSupB and its parent cSupB, so each cSupB has only one parent cSupB with a topological distance of 1. Therefore, all cSupBs in the area determined by a pair of source/sink nodes containing all samples, except for the root cSupB that has no parent node, all other cSupBs have and only have one adjacent parent cSupB. If there are n cSupBs, then the constructed cSupB topology structure has n-1 edges, and because each cSupB can continuously trace its parent cSupB until root cSupB, so connectivity is held. In summary, the cSupB topology structure is a tree.

**Property 5**: If the traversal is finished in advance, then at least one half-visited node can be obtained, and at least one is on the cycle.

Proof: According to the full coverage theorem, when the traversal is finished in advance, it means that there is a cycle on the graph.

Traversing the nodes have been visited from the start node along the direction of the edge, and at the same time traversing the nodes unvisited from the end node against the direction of the edge. The node at the junction must be half-visited because if it is a visited state node, we can continue to visit along the edge and its outgoing node cannot be unvisited. If it is the unvisited state, then its incoming node cannot be visited. Therefore, there is at least one half-visited state node.

Also, analyzing the reason for the appearance of half-visited nodes. The reason for the occurrence of type I half-visited state node is that the incoming nodes cannot be fully visited due to the cycle, while the type II half-visited state node is produced because of the type I half-visited state. Therefore, there must be half-visited nodes on the cycle.

**Property 6**: Let $V_{0}$ denote the set of all half-visited nodes when encountering a cycle. If existing a node$u$ who is the parent node of other half-visited nodes, it must be a part of the cycle.

Proof: By contradiction, suppose there is a half-visited node $u$ that is the parent node of other half-visited state nodes, and $u$ is not part of the cycle. Selecting all nodes $v$ satisfying $color\left( u \right)\cap color\left( v \right)\neq\emptyset$ from the start node to $u$ forming a subgraph $G_{1}$, which is a directed acyclic graph. By the full coverage theorem, we know that all nodes can be visited, which contradicts with that $u$ is half-visited and the hypothesis does not hold.

**Property 7**: Let $V_{0}$ denote the set of all half-visited nodes when encountering a cycle. If the half-visited nodes only involve one cycle, then starting from a half-visited node $u$ on the cycle, we can obtain other half-visited nodes and $u$ itself.

Proof: If we start to visit from a half-visited state node $u$ on the cycle, similar to fig. 3 in the paper, it is equivalent to transform the cyclic graph into an acyclic graph. According to the full coverage theorem, we can know that we can visit all nodes, of course, can also visit other half-visited nodes. Because one incoming edge of $u$ has not been visited yet, it can still be visited by itself.

# 2. Traversal and matching

**Nodes’ four visiting states**

Before traversing the graph, we must label the node visiting state. For each node $u$, we label four visiting states:

-1: unvisited (no adjacent incoming node of $u$ is visited)

0: half-visited (at least one adjacent incoming node of $u$ is visited and at least one adjacent incoming node of $u$ is unvisited)

1: to-be-visited (all the adjacent incoming nodes of $u$ are visited, but $u$ is unvisited and can be visited anytime)

2: fully visited ($u$ and its adjacent incoming nodes are all visited)

| Algorithm 1: Graph decomposition and reorganization (Postorder-like traversal, cSupB obtaining and cycle identification) | |
| --- | --- |
| Input: Colorset colors, start node $u^{'}$, end node $v^{'}$ | Traversal is against the direction of the edge from the end node |
| 1. function Get_cSupB(colors, $v^{'}$) | colors $\leftarrow$ color vector of all edges |
| 1. $S$.push($v^{'}$) | $S$ $\leftarrow$ to-be-visited nodes |
| 1. visited[$v^{'}$] $\leftarrow$ 2 | Visiting states: unvisited (-1), half-visited (0), to-be-visited (1) and fully visited (2) |
| 1. VisitEnd $\leftarrow$ false |  |
| 1. Offset[$v^{'}$] $\leftarrow$ 1 | Offset $\leftarrow$ node’s offset value |
| 1. RefNodePos[$v^{'}$] $\leftarrow$ 1 |  |
| 1. while VisitEnd = false do |  |
| 1. while $S$ $\neq\emptyset$ do |  |
| 1. $u$ $\leftarrow$ $S$[1] |  |
| 1. if $u\in HV$ then remove $u$ from $HV$ | $HV$ $\leftarrow$ half-visiting nodes |
| 1. for each $w\in{IN}_{u}$ do | ${IN}_{u}$ $\leftarrow$ all the adjacent incoming nodes of $u$ |
| 1. if visited[$w$] = 2 then | Meet cycle start node $w$ |
| 1. CNI.push([$u,w$]) | CN $\leftarrow$ cycle node intervals |
| 1. continue |  |
| 1. if exist $w^{'}\in{ON}_{w}$ is not fully visited then | ${ON}_{w}$ $\leftarrow$ all the adjacent outgoing nodes of $w$ |
| 1. visited[$w$]$\leftarrow$ 1 |  |
| 1. $HV$.push($w$) |  |
| 1. continue |  |
| 1. if $w$’s indegree ind > 2 then |  |
| 1. $Q$.push($w$) | $Q \leftarrow$ node queue |
| 1. if $w$’s outdegree outd > 2 then |  |
| 1. for $i$length($Q$):1 do |  |
| 1. $v$ $\leftarrow$ $Q$[$i$] |  |
| 1. if ColorCMP(${OE}_{w},{IE}_{v}$) = true then | ${OE}_{w}({IE}_{v})$ $\leftarrow$ all the adjacent outgoing (incoming) edges of $w$($v$) |
| 1. $c_{0}\leftarrow$ NodeColor(colors,$v$) $\cap$NodeColor(colors,$w$) |  |
| 1. CSUPB.push(<$w,v, c_{0}$>) | CSUPB $\leftarrow$ cSupBs |
| 1. if NodeColor($v$) $\subseteq$ NodeColor($w$) |  |
| 1. remove $v$ from $Q$ |  |
| 1. break |  |
| 1. $S$.push($w$) |  |
| 1. visited[$w$]$\leftarrow$ 2 |  |
| 1. find $w_{0},w_{1}\in{ON}_{w}$ do |  |
| 1. ${MaxO}_{w}$ $\leftarrow$ Offset[$w_{0}$] | ${MaxO}_{w}$ $\leftarrow$ maximum offset value of nodes in ${ON}_{w}$ |
| 1. ${MinO}_{w}$ $\leftarrow$ Offset[$w_{1}$] | ${MinO}_{w}$ $\leftarrow$ minimum offset value of nodes in ${ON}_{w}$ |
| 1. Offset[$w$] $\leftarrow{MaxO}_{w}$+1 |  |
| 1. Node2Gap[$w$] $\leftarrow{MaxO}_{w}$ -${MinO}_{w}$ | Node2Gap $\leftarrow$ gap length |
| 1. Node2GapColor[$w$] $\leftarrow$ NodeColor($w_{1})$ | Node2GapColor $\leftarrow$ gap color |
| 1. if existing $w_{2}\in{ON}_{w}$ & RefNodePos[$w_{2}]$ exists then |  |
| 1. RefNodePos[$w$] $\leftarrow$ RefNodePos[$w_{2}]$ + 1 |  |
| 1. if $HV$=$\emptyset$ then VisitEnd = true | There is no cycle |
| 1. else |  |
| 1. find $x\in HV$ whose offset value is minimum do |  |
| 1. visited[$x$] $\leftarrow2$ |  |
| 1. $S$.push($x$) |  |
| 1. MaxOffset $\leftarrow$ Offset[$u^{'}$] |  |
| 1. MaxRefPos $\leftarrow$ RefNodePos[$u^{'}$] |  |
| 1. for each $w^{'}\in DBG$ do |  |
| 1. Offset[$w^{'}$] $\leftarrow$ MaxOffset - Offset[$w^{'}$] + 1 |  |
| 1. RefNodePos[$w^{'}$] $\leftarrow$ MaxRefPos - RefNodePos[$w^{'}$] + 1 |  |
| 1. return CSUPB,Offset, RefNodePos, CNI, Node2Gap, Node2GapColor |  |

| Algorithm 2: Get edge color |  |
| --- | --- |
| Input: Colorset colors, edge $e^{'}$ |  |
| 1. function EdgeColor(colors,$e^{'}$) |  |
| 1. for $i$1: N do | N $\leftarrow$ sample number |
| 1. $c$ $\leftarrow$ colors[N * $e^{'}$ + $i$] |  |
| 1. $s$ $\leftarrow$ $s$ + ‘$c$’ | $s$ $\leftarrow$ color string |
| 1. return $s$ |  |

| Algorithm 3: Get node color |  |
| --- | --- |
| Input: Colorset colors, Node $u$ |  |
| 1. function NodeColor(colors,$u$) |  |
| 1. for each $e^{'}\in{IE}_{u}$ do | ${IE}_{u}$ $\leftarrow$ the adjacent incoming edges of u |
| 1. $s$ $\leftarrow s$ $\cup$ EdgeColor(colors, $e^{'}$) | $s$ $\leftarrow$ color string |
| 1. return $s$ |  |

| Algorithm 4: Color matching principle |  |
| --- | --- |
| Input: Colorset colors, Outgoing edge set OE, incoming edge set IE |  |
| 1. function ColorCMP(colors, OE, IE) |  |
| 1. for each $e^{'}\in OE$ do |  |
| 1. $s^{'}$ $\leftarrow$ $s^{'}$ $\cup$ Getedgecolor(colors, $e^{'}$) | $s^{'}$ $\leftarrow$ color string |
| 1. for each $e^{''}\in IE$ do |  |
| 1. $s^{''}$ $\leftarrow$ $s^{'}'$ $\cup$ Getedgecolor(colors, $e^{''}$) | $s^{''}$ $\leftarrow$ color string |
| 1. count1$\leftarrow$ 0, count2$\leftarrow$ 0 |  |
| 1. for each $e^{'}\in OE$ do |  |
| 1. if Getedgecolor(colors, $e^{'}$) $\cap s^{''}\neq\emptyset$ then |  |
| 1. count1++ |  |
| 1. for each $e^{''}\in IE$ do |  |
| 1. if Getedgecolor(colors, $e^{'}'$) $\cap s^{'}\neq\emptyset$ then |  |
| 1. count2++ |  |
| 1. if count1>1 & count2 >1 then return true |  |
| 1. else return false |  |

| Algorithm 5: cSupBs’ subordination |  |
| --- | --- |
| Input: cSupB ordering set Order, cSupB color set Bubcolor |  |
| 1. function Bub_Relation(Order,Bubcolor) |  |
| 1. for $i$1: bubnum do | bubnum $\leftarrow$ number of cSupBs |
| 1. $c_{1}$ $\leftarrow$ Bubcolor[$i$] |  |
| 1. if $c_{1}=C$ then | $C$ $\leftarrow$ color string that contains all samples |
| 1. BubParent[$i$] $\leftarrow$ -1 | BubParent $\leftarrow$ cSupB’s nearest parent cSupB |
| 1. continue |  |
| 1. for $j$ $i+1$: bubnum |  |
| 1. $c_{2}$ $\leftarrow$ Bubcolor[$j$] |  |
| 1. if $c_{1}$ = $c_{1}\cap c_{2}$ & $c_{1}\neq c_{2}$ then |  |
| 1. BubParent[$i$] $\leftarrow$ $j$ |  |
| 1. BubChild[$j$].push($i$) | BubChild $\leftarrow$ all the cSupB’s nearest child cSupBs |
| 1. break |  |
| 1. return BubParent, BubChild |  |

# 3. Output file format

(1) Cycle Cut Position Format（CCP）

File format: *count type start length cutpos thick rate label_color*

Here,

1) *count* represents the ordering id of the cutting area

2) *type* indicates the type of cutting area. We divide the cutting nodes into three categories:

I. The bridge. This type is our ideal cutting point;

II. Not the bridge but involving all samples;

III. Not the bridge and involving only a portion of the samples.

3) *start* represents the start node of the area, *length* represents the length of the area, and *cutpos* represents the cutting position. Generally, the middle position of the cutting area is selected.

4) *thick* represents the thickness of the position. *rate* represents $R=L/t$, $L$ represents the length, and $t$ represents thickness. The longer $L$ and the smaller $t$, the better. *label_color* represents the color of the cut segment.

(2) Colored-superbubble topology（**CST**）

CST file format to store cSupB topology information, the format is as follows:

*bubid bublevel sourcepos sinkpos sourcenode:sinknode fatherbub_id:childbub_id sourcegap sourcevar bubstructure bubcolor*

Here,

1) *bubid* and *bubstructure* are two named methods of cSupB. *bubid* is an integer of 0, 1, 2... which is the ordering that cSupB obtained. According to the traversal strategy and the cSupB matching method, the *bubid* of the child cSupB is smaller than any parent cSupB; *bubstructure* is the representation of the topological structure of the cSupB in the graph. For example, bub22_3_1 means that the current bubid is 1, which is the child bubble of cSupB 3, and cSupB 3 is the child bubble cSupB 22. cSupB 22 is the root node of the cSupB tree and contains all samples. The bubble structure also contains the information of the bubble level, and bub22_3_1 is a bubble with a hierarchical depth of 3 (bublevel=3).

2) *bublevel* has been mentioned in 1), which means the hierarchical depth of cSupB in the cSupB tree. The hierarchical depth of the root cSupB is 1. The larger the level value, the more complex the graph structure is.

3) *sourcepos* and *sinkpos* are the offset values of sourcenode and sinknode, respectively. Then *pos(source)-pos(sink)+1* is the span of cSupB.

4) *fatherbub_id: childbub_id* represents the bubid of the nearest parent cSupB and all child cSupBs. According to our previous definition, there is only one fatherbub, and there can be more than one childbub, separated by a semicolon. If it does not exist, it is recorded as -1. The root cSupB has no fatherbub and the leaf node has no childbub.

5) *bubcolor* represents the color of cSupB. According to our definition, it is the intersection of sourcenode and sinknode colors. The representation is a 0-1 string of length s, and s is the number of all samples.

6) *sourcegap* and *sourcevar* respectively represent the number of gaps and variant types at the source node. In the colored de Bruijn graph we constructed, the occurrence of variant corresponds to the generation of the source node almost one-to-one, so the variant is discussed near the source node and it is the key idea of our node-based variant detection method.

In the process of traversing the graph, we first define a variable of node2gap and node2gapcolor to save the maximum number of gaps and their colors near the source node. Then after determining the cSupB inclusion relationship, using this existing information to determine the type of variant at each source, and finally mapping the variant to each base to obtain the final variant analysis result. There are four types of variant: 1-SNP, 2-Delete, 3-Insert, 4-Indel. Since we have introduced a reference genome when constructing the graph, when analyzing insertion or deletion variants, its priority is to compare with the reference genome. Details can be seen in part4 “Node-based variant analysis”.

(3) Colored-superbubble detailed information (CSDI) format

In addition to files in CST format, if we want to obtain more detailed information about a certain cSupB, we can choose to output a more detailed file called CSDI format. Besides saving some CST information, CSDI also added *node label/color, node/edge/branch/supernode, self-defined indexes, and all branch detailed information*. Here is only a brief introduction to *self-defined indexes*.

Self-defined indexes

This part is some of the characteristic description indicators of cSupB.

a. density

$$density=(e-(n-1))/(4n-(n-1))=(e-n+1)/(3n+1)$$

Due to the sparseness of the actual graph, 4n can be reduced to 2n, then

$$density=(e-(n-1))/(2n-(n-1))=(e-n+1)/(n+1)$$

b. thickness

$thick=(n+2)/l$，here thick≥2 mostly

(4) Node nearby information (NNI) and offset value information (OVI)

Finally, we also output some necessary auxiliary files to store node nearby information (NNI) and offset value information (OVI).

NNI:

*node label color*

*incoming nodes & colors*

*outgoing nodes & colors*

OVI:

*node nodepos refnodepos maxgap*

Here, *nodepos* and *refnodepos* represent the offset value in the graph and reference genome respectively. If a node cannot be obtained in the reference, we use -1 to represent it.

# 4. Variant detection method

**Father-Children Variant Detection（FCVD）：**

**Condition1**:node2var[a] exists，and bubble involves reference (fig. 2a)，$ref\_ins$=false initially.

1. If var(a)= SNP/Del
2. gap1=0, var(b)=SNP;
3. gap1>0, var(b)=DEL;
4. If var(a)= Ins
5. gap1=0, var(b)=SNP;
6. gap1>0, var(b)=Indel, $ref\_ins$=true;


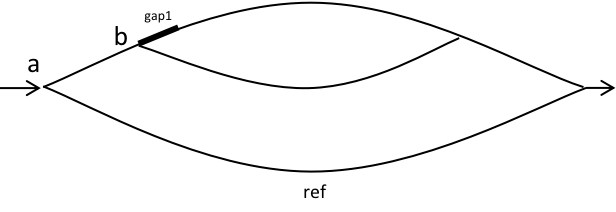

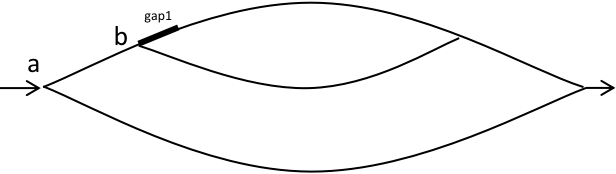


a b

Fig. 2 Two conditions of Father-Children Variant Detection algorithm

**Condition 2**: node2var[a] exists, and bubble doesn’t involve reference (fig. 4b).

1. If var(a)=SNP/DEL
2. gap1=0, var(b)=SNP;
3. gap1>0 & $ref\_ins$=true, var(b)=Indel;
4. gap1>0 & $ref\_ins$=false, var(b)=DEL;
5. If var(a)=INS/Indel
6. gap1=0, var(b)=SNP;
7. gap1>0, var(b)=Indel;

Finally,

if var(b)=SNP/Del, node2actualgap[b]=node2gap[b];

if var(b)=Ins/Indel, node2actualgap[b] = -node2gap[b] (‘-’ means gap is unsure)

The determination of each source variant type here is determined based on the variant type of its nearest parent source node. Compared with the reference genome involved in step 1, the strategy for determining the variant here is not perfect. For the variants that are difficult to determine whether it is Del or Ins, we use Indel to express. Besides, there is not much correction to the number of gaps at the source node and we use a negative value representing the gap of the node whose variant type is uncertain. Similarly, during mapping from the graph to the reference genome in RPD, there may be multiple nodes mapped to the same location simultaneously or different variants have intersections. In addition to the four types of variants 1: SNP, 2: Del, 3: Ins, 4: Indel, if different variants are mapped to the same locus, setting the locus’ variant value to be the negative of the sum of the variant values. For example, if a locus has both SNP and Del variants, then the locus variant value is $-(1+2)=-3.$

| Algorithm 6: Nested Variant Detection (NVD-1) |  |
| --- | --- |
| Input: CSUPB, Node2Gap, Node2GapColor |  |
| 1. function NVD1(CSUPB, Node2Gap, Node2GapColor) |  |
| 1. INScond $\leftarrow$ false |  |
| 1. INSgap $\leftarrow$ 0 |  |
| 1. INScolor $\leftarrow$ 0 |  |
| 1. for $i$ 1: bubnum do |  |
| 1. $<s,t,c> \leftarrow$ CSUPB[$i$] |  |
| 1. if $s\in REF$ & Node2Var[$s$] doesn’t exist then | REF $\leftarrow$ node set produced by reference genome |
| 1. $gc$ $\leftarrow$ Node2GapColor[$s$] |  |
| 1. $g$ $\leftarrow$ Node2Gap[$s$] |  |
| 1. if $g=0$ then Node2Var[$s$] $\leftarrow$ 1 | Node2Var $\leftarrow$ node variant |
| 1. else |  |
| 1. if INDcond = false then |  |
| 1. Node2ActualGap[$s$] $\leftarrow$ $g$ | Node2ActualGap $\leftarrow$ adjusted gap |
| 1. if $gc$ contains reference then |  |
| 1. Node2Var[$s$] $\leftarrow$ 3 |  |
| 1. INScond $\leftarrow$ true |  |
| 1. INSgap $\leftarrow$ $g$ |  |
| 1. INScolor $\leftarrow$ NodeColor($s$) |  |
| 1. else Node2Var[$s$] $\leftarrow$ 2 | $gc$ doesn’t contain reference genome |
| 1. else | INDcond = true |
| 1. if $gc$ doesn’t contain reference then |  |
| 1. CapColor $\leftarrow$ INScolor $\cap$ $gc$ |  |
| 1. INScolor $\leftarrow$ INScolor $\cup$ NodeColor[$s$] |  |
| 1. if CapColor = 0 then |  |
| 1. if INSgap < $g$ then |  |
| 1. Node2Var[$s$] $\leftarrow$ 2 |  |
| 1. Node2ActualGap[$s$] $\leftarrow$ $g$ - INSgap |  |
| 1. else if INSgap = $g$ then |  |
| 1. Node2Var[$s$] $\leftarrow$ 1 |  |
| 1. Node2ActualGap[$s$] $\leftarrow$ 0 |  |
| 1. else Node2Var[$s$] $\leftarrow$ 3 |  |
| 1. Node2ActualGap[$s$] $\leftarrow$INSgap - $g$ |  |
| 1. else Node2Var[$s$] $\leftarrow$ 2 | CapColor $\neq$ 0 |
| 1. Node2ActualGap[$s$] $\leftarrow$ $g$ |  |
| 1. else Node2Var[$s$] $\leftarrow$ 3 | $gc$ doesn’t contain reference genome |
| 1. Node2ActualGap[$s$] $\leftarrow$ $g$ |  |
| 1. INScolor $\leftarrow$ INScolor $\cup$ NodeColor($s$) |  |
| 1. INSgap += $g$ |  |
| 1. if INScolor = C & NodeColor($s$) = C then | $C$ $\leftarrow$ color string that contains all samples |
| 1. INScond $\leftarrow$ false |  |
| 1. INSgap $\leftarrow$ 0 |  |
| 1. INScolor $\leftarrow$ 0 |  |
| 1. return Node2Var, Node2ActualGap |  |
|  |  |

| Algorithm 7: Nested Variant Detection (NVD-2) |  |
| --- | --- |
| Input: CSUPB, Node2Gap, Node2GapColor, BubParent | |
| 1. function NVD2(CSUPB, Node2Gap, Node2GapColor) |  |
| 1. INScond $\leftarrow$ false |  |
| 1. INSgap $\leftarrow$ 0 |  |
| 1. INScolor $\leftarrow$ 0 |  |
| 1. for $i$ 1: bubnum do |  |
| 1. $<s,t,c> \leftarrow$ CSUPB[$i$] |  |
| 1. if Node2Var[$s$] exists then | REF $\leftarrow$ node set produced by reference genome |
| 1. $gc$ $\leftarrow$ Node2GapColor[$s$] | $gc$ $\leftarrow$ gap color |
| 1. $g$ $\leftarrow$ Node2Gap[$s$] |  |
| 1. if $g=0$ then Node2Var[$s$] $\leftarrow$ 1 | Node2Var $\leftarrow$ node variant |
| 1. Node2ActualGap[$s$] $\leftarrow$ 0 |  |
| 1. else $pb$ $\leftarrow$ $i$ | $pb$ $\leftarrow$ parent bubble id |
| 1. $S$.push($s$) | $S$ $\leftarrow$ temporary set storing source nodes |
| 1. $ps$ $\leftarrow$ $s$ | $ps$ $\leftarrow$ parent source node |
| 1. $<s^{'},t^{'},c^{'}> \leftarrow$ CSUPB[$pb$] |  |
| 1. while Node2Var[$s^{'}$] doesn’t exists do |  |
| 1. if $s^{'}\neq s$ then $S$.push($s^{'}$) |  |
| 1. $ps$ $\leftarrow s^{'}$ |  |
| 1. $pb$ $\leftarrow$ BubParent[$pb$] |  |
| 1. $<s^{''},t^{''},c^{''}> \leftarrow$ CSUPB[$pb$] |  |
| 1. $s^{'}$ $\leftarrow s^{''}$ |  |
| 1. $ps$ $\leftarrow s^{'}$ |  |
| 1. $l$ $\leftarrow$ $S$.size() |  |
| 1. ref_ins $\leftarrow$ false |  |
| 1. for $j$ $l$:1 do |  |
| 1. $cs$ $\leftarrow$ $S$[$j$] | $cs$ $\leftarrow$ child source node |
| 1. $cg$ $\leftarrow$ Node2Gap[$cs$] | $cg$ $\leftarrow$ child source gap |
| 1. $pv$ $\leftarrow$ Node2Var[$ps$] | $pv$ $\leftarrow$ parent source variant |
| 1. if $cg$ = 0 then |  |
| 1. $cv$ $\leftarrow$ 1 | $cv$ $\leftarrow$ child source node variant |
| 1. Node2ActualGap[$cs$] $\leftarrow$ 0 |  |
| 1. $ps$ $\leftarrow$ $cs$ |  |
| 1. continue |  |
| 1. if $j=l$ then |  |
| 1. if $fv$< 2 then |  |
| 1. $cv$ $\leftarrow$ 2 |  |
| 1. Node2ActualGap[$cs$] $\leftarrow$ Node2Gap[$cs$] |  |
| 1. else $cv$ $\leftarrow$ 4 |  |
| 1. Node2ActualGap[$cs$] $\leftarrow$ -Node2Gap[$cs$] |  |
| 1. ref_ins $\leftarrow$ true |  |
| 1. else |  |
| 1. if $fv$< 2 then |  |
| 1. if ref_ins = true then |  |
| 1. $cv$ $\leftarrow$ 4 |  |
| 1. Node2ActualGap[$cs$] $\leftarrow$ -Node2Gap[$cs$] |  |
| 1. else $cv$ $\leftarrow$ 2 |  |
| 1. Node2ActualGap[$cs$] $\leftarrow$ Node2Gap[$cs$] |  |
| 1. else $cv$ $\leftarrow$ 4 |  |
| 1. Node2ActualGap[$cs$] $\leftarrow$ -Node2Gap[$cs$] |  |
| 1. Node2Var[$cs$] $\leftarrow$ $cv$ |  |
| 1. $ps$ $\leftarrow$ $cs$ |  |
| 1. return Node2Var, Node2ActualGap |  |

| Algorithm 8: Reference Position Determination (RPD) |  |
| --- | --- |
| Input: CSUPB, Node2Var, Node2ActualGap, BubParent, RefNodePos, Offset | |
| 1. function RPD(CSUPB, Node2Var, Node2ActualGap, BubParent, RefNodePos, Offset) |  |
| 1. for $i$ 1: bubnum do |  |
| 1. $<s,t,c> \leftarrow$ CSUPB[$i$] |  |
| 1. if RefNodePos[$s$] doesn’t exist then | REF $\leftarrow$ node set produced by reference genome |
| 1. $pb$ $\leftarrow$ $i$ | $pb$ $\leftarrow$ parent bubble id |
| 1. $S$.push($s$) | $S$ $\leftarrow$ temporary set storing source nodes |
| 1. $ps$ $\leftarrow$ $s$ | $ps$ $\leftarrow$ parent source node |
| 1. $<s^{'},t^{'},c^{'}> \leftarrow$ CSUPB[$pb$] |  |
| 1. while RefNodePos[$s^{'}$] doesn’t exists do |  |
| 1. if $s^{'}\neq s$ then $S$.push($s^{'}$) |  |
| 1. $ps$ $\leftarrow s^{'}$ |  |
| 1. $pb$ $\leftarrow$ BubParent[$pb$] |  |
| 1. $<s^{''},t^{''},c^{''}> \leftarrow$ CSUPB[$pb$] |  |
| 1. $s^{'}$ $\leftarrow s^{''}$ |  |
| 1. $ps$ $\leftarrow s^{'}$ |  |
| 1. $l$ $\leftarrow$ $S$.size() |  |
| 1. for $j$ $l$:1 do |  |
| 1. $cs$ $\leftarrow$ $S$[$j$] | $cs\leftarrow$ child source node |
| 1. $pg$ $\leftarrow$ Node2ActualGap[$ps$] | $pg\leftarrow$ parent node gap |
| 1. $cg$ $\leftarrow$ Node2ActualGap[$cs$] | $cg\leftarrow$ child node gap |
| 1. $pv$ $\leftarrow$ Node2Var[$ps$] | $pv\leftarrow$ parent source node variant |
| 1. $d$ $\leftarrow$ Offset[$ps$] – RefNodePos[$ps$] |  |
| 1. if $pv$> 2 then |  |
| 1. if $pv$> 3 then |  |
| 1. if $pg$ > $cg$ then |  |
| 1. $gd$ $\leftarrow pg$ - $cg$ |  |
| 1. RefNodePos[$cs$] $\leftarrow$ - (Offset[$cs$] - $d$ - $gd$) |  |
| 1. else |  |
| 1. $gd$ $\leftarrow cg$ - $pg$ |  |
| 1. RefNodePos[$cs$] $\leftarrow$ - (Offset[$cs$] – $d$+ $gd$) |  |
| 1. else |  |
| 1. $gd$ $\leftarrow pg$ - $cg$ |  |
| 1. RefNodePos[$cs$] $\leftarrow$ Offset[$cs$] - $d$ - $gd$ |  |
| 1. else |  |
| 1. RefNodePos[$cs$] $\leftarrow$ Offset[$cs$] - $d$ |  |
| 1. NodePos2RefPos[Offset[$cs$]] $\leftarrow abs$(RefNodePos[$cs$]) | NodePos2RefPos $\leftarrow$ node position to reference position |
| 1. RefPos2NodePos$[abs$(RefNodepos[$cs])] \leftarrow$Offset[$cs$] | RefPos2NodePos $\leftarrow$ reference position to node position |
| 1. $ps$ $\leftarrow cs$ |  |
| 1. return NodePos2RefPos, RefPos2NodePos, RefNodePos |  |

# 5 Simulation method

Steps:

(1) Prepare data. Before the simulation, we need to select a reference genome sequence, remove or modify the degenerate bases, and calculate the sequence length $L$; then the following variables need to be preset: variant type $(var1,var2,...,varx)$ and its number $(n1,n2,...,nx)$, the number of simulated samples $s$, the maximum length of indel and insertion variant *maxdel* and *maxins*;

(2) Determine the location and variant type. Randomly select an integer value between (0, L) as the variant site, and randomly select an integer value between $(0, n1+n2+...+nx)$to determine variant type. Repeat this step to simulate $n1+n2+...+nx$ times, and perform sorting and deduplication operations on the set of variant sites obtained;

(3) Random produce variant information. The initial values ​​of $s$ samples are all reference genomes, given a site, then randomly determine the variant information.

a. For each position, a random integer s' between $[1, s-1]$ is set as the number of samples that undergo variant;

b. Randomly select $s'$ integers from $[1, s-1]$ as the mutated sample id;

c. Determine the variant result. If it is an SNP, randomly a base that is different from the reference genome site. If it is del, random an integer in $[1,maxdel]$ as the gap length, and if it is ins, randomly an integer in $[1,maxins]$ as the insertion length;

(4) Obtain the simulation sequence. Start traversing from large to small, repeat step (3) for each location, and replace the randomly selected samples’ original base with the randomly obtained variant result to obtain the final simulation sequence.

In the entire simulation process, there are four prerequisites: reference genome, variant type and quantity, sample number, the maximum length of del/ins. And six random processes: determination of the variant location, the variant type, the variant result, the mutation sample number, the mutated samples, and the del/ins length. Here, the $s-th$ sequence we simulated is the reference sequence and $maxdel=maxins=1$.

# 6 Calculation result

Here precision and recall rate are calculated by $typemaped\_var$ whose rates are a few lower compared with $locationmapped\_var$.
